# Supplementary material for: Knowledge, perceptions, and practices of dental professionals and students regarding obturation in endodontic procedures: a nationwide cross-sectional survey
Source: PeerJ. 2025 Jun 3;13:e19455. doi: 10.7717/peerj.19455 (PMC12143286; doi:10.7717/peerj.19455)
Supplement: Supplemental Information 2 [file peerj-13-19455-s002.docx]

**Trends of Obturation in Endodontic Procedure**

**Demographic Characteristics**

**1. Gender:**

1. Male
2. Female

**2. Age in years:**

**3. Current Academic position:**

1. 1 st BDS
2. 2 nd BDS
3. 3 rd BDS
4. 4 th BDS
5. Intern
6. Post Graduate students
7. Faculty in dental school
8. Private Practice

**Knowledge Section**

**1. What is the primary purpose of root canal obturation in endodontics?**

1. To remove bacteria from the root canal system
2. To shape the root canal
3. To fill and seal the root canal space
4. To extract the tooth

**2. When to obturate the root canal?**

1. When tooth is asymptomatic
2. Canal should properly dry
3. No pathological radiographic finding
4. Any other criteria (your answer)

**3. Which material is commonly used for obturation in endodontics?**

1. Amalgam
2. Composite resin
3. Gutta-percha and sealer
4. Silver points

**4. What are the ideal properties of an obturating material?**

1. Sealing ability, biocompatibility, and dimensional stability
2. Radiopacity, flowability, and setting time
3. Antibacterial properties, removability, and flowability
4. Biocompatibility, radiopacity, and setting time
5. All of the above

**5. What is the purpose of using an endodontic sealer in obturation?**

1. To enhance the flowability of the obturating material
2. To create a mechanical bond with the tooth structure
3. To fill the gaps between the gutta-percha and the canal walls
4. To disinfect the root canal system
5. Multiple Options Selected

**6.** **What are the different techniques for obturation?**

1. Cold lateral compaction
2. Warm lateral compaction
3. Continuous wave compaction
4. Thermoplasticized gutta percha injection
5. Carrier based gutta percha injection
6. Thermomechanical compaction technique
7. Chemically plastered gutta percha obturation
8. Custom cone obturation technique
9. Multiple Techniques Selected

**7. How does obturation contribute to the success of root canal treatment?**

1. It prevents bacterial recontamination, prevents root resorption, and promotes healing
2. It eliminates the need for postoperative medications
3. It ensures complete removal of pulp tissue
4. None of the above
5. Multiple Options Selected

**8. What are the potential complications associated with inadequate obturation?**

1. Tooth discoloration and bad breath
2. Root fracture and postoperative pain
3. Gum recession and tooth sensitivity
4. Enamel erosion and excessive salivation
5. Multiple Complications from the above.

**9. Which form of Gutta percha cone you used in clinics?**

1. Alpha form
2. Beta form
3. Active gutta percha
4. Any of the above

**10. Which method did you use to disinfect gutta percha cone?**

- 1. Heat sterilization
  2. 5.25% Sodium hypochlorite for one minute
  3. 2% glutaraldehyde
  4. 2% Chlorhexidine

**Attitude Section**

**1. How confident do you feel about your knowledge and skills in root canal obturation?**

- 1. Very confident
  2. Somewhat confident
  3. Not very confident
  4. Not confident at all

**2. How important do you consider obturation to be in the overall success of root canal treatment?**

- 1. Extremely important
  2. Moderately important
  3. Slightly important
  4. Not important

**3. Are you open to adopting new obturation techniques or materials in your practice?**

- 1. Yes, always eager to try new methods
  2. Yes, if supported by scientific evidence
  3. No, prefer sticking to traditional techniques
  4. No, not interested in exploring new options

**4. How often do you attend continuing education courses or workshops related to root canal obturation?**

- 1. Regularly, at least once a year
  2. Occasionally, every few years
  3. Rarely, only when required
  4. Never, I do not attend any courses or workshops

**5. How willing are you to invest in new obturation materials or equipment for your practice?**

- 1. Very willing, I prioritize staying up-to-date
  2. Somewhat willing, if it proves to be beneficial
  3. Not very willing, I prefer cost-effective options
  4. Not willing at all, I stick to what I already have

**6. Do you believe that advancements in obturation techniques have improved the success rates of root canal treatment?**

- 1. Yes, significantly
  2. Yes, to some extent
  3. No, success rates have remained the same
  4. No, they have worsened the outcomes

**7. How comfortable are you in handling complex root canal anatomy during obturation procedures?**

- 1. Very comfortable, it is one of my strengths
  2. Somewhat comfortable, with some difficulty
  3. Not very comfortable, it poses challenges for me
  4. Not comfortable at all, I avoid complex cases

**8. Are you satisfied with the current obturation materials available in the market?**

- 1. Yes, completely satisfied
  2. Somewhat satisfied, but improvements are needed
  3. Not very satisfied, there is room for better options
  4. Not satisfied at all, I am dissatisfied with the available materials

**9. Do you believe that obturation techniques should be standardized for better consistency and outcomes?**

- 1. Yes, standardization is crucial for improved results
  2. Yes, but with room for individual adaptation
  3. No, diversity in techniques is beneficial
  4. No, it is not necessary for better outcomes

**10. How confident are you in educating your patients about the importance of obturation in root canal treatment?**

- 1. Very confident, I effectively communicate its significance
  2. Somewhat confident, but I could improve my communication
  3. Not very confident, I struggle to convey its importance
  4. Not confident at all, I do not discuss it with my patients

**Practice Section**

**1. Which obturation technique do you primarily use in your daily practice?**

- 1. Lateral condensation
  2. Vertical compaction
  3. Single cone technique
  4. Carrier-based systems

**2. Do you routinely use a rubber dam during obturation procedures?**

- 1. Yes, for all cases
  2. Yes, for most cases
  3. Occasionally, for certain cases
  4. No, I do not use a rubber dam

**3. How often do you encounter difficulties in achieving a satisfactory obturation in root canal treatments?**

- 1. Rarely, I seldom encounter difficulties
  2. Occasionally, it happens from time to time
  3. Frequently, it is a common challenge for me
  4. Always, I consistently face difficulties

**4. Are you satisfied with the current obturation techniques you use in terms of efficiency and predictability?**

- 1. Yes, completely satisfied
  2. Somewhat satisfied, but improvements are needed
  3. Not very satisfied, there are limitations to overcome
  4. Not satisfied at all, I am dissatisfied with the techniques

**5. How often do you evaluate the quality of your obturations using radiographs or other imaging techniques?**

- 1. Always, after every obturation procedure
  2. Often, but not after every procedure
  3. Rarely, only in certain cases
  4. Never, I do not evaluate the obturations

**6. Do you document the obturation technique and materials used in patient records?**

- 1. Always, it is a standard practice for me
  2. Often, but not consistently
  3. Rarely, only when necessary
  4. Never, I do not document the obturation details

**7. How frequently do you perform retreatment procedures for failed root canal obturations?**

- 1. Rarely, retreatments are infrequent
  2. Occasionally, for a few cases
  3. Frequently, it is a common procedure for me
  4. Always, retreatments are the majority of my cases

**8. How often do you use bioceramic sealers in your obturation procedures?**

- 1. Always, it is my preferred sealer
  2. Often, for specific cases
  3. Rarely, only when requested by patients
  4. Never, I do not use bioceramic sealers

**9. Do you actively seek feedback from patients regarding their experience with root canal obturation?**

- 1. Yes, I regularly ask for patient feedback
  2. Sometimes, if patients express concerns
  3. Rarely, I do not actively seek feedback
  4. Never, I do not consider patient feedback important

**10. How often do you update your knowledge and skills in modern root canal obturation techniques?**

- 1. Regularly, through continuous education and training
  2. Occasionally, when new techniques emerge
  3. Rarely, I rely on my existing knowledge and skills
  4. Never, I do not invest in updating my knowledge and skills
